# Sex differences in the effects of shift work-like schedules on gut microbiome and intestinal barrier in relation to stroke survival

**DOI:** 10.1371/journal.pone.0355842

**Published:** 2026-08-14

**Authors:** Ella Barnum, Jonathan L. Turck, Karienn A. Souza, Kathiresh Kumar Mani, Rachel Pilla, Amutha Selvamani, Farida Sohrabji, David J. Earnest

**Affiliations:** 1 Department of Neuroscience and Experimental Therapeutics, Texas A&M Health Science Center, College of Medicine, Bryan, Texas, United States of America; 2 Department of Small Animal Clinical Sciences, Texas A&M University, College of Veterinary Medicine, College Station, Texas, United States of America; 3 Department of Veterinary Medicine and Animal Sciences, University of Milan, Lodi, Italy; 4 Women’s Health in Neuroscience Program, Texas A&M Health Science Center, College of Medicine, Bryan, Texas, United States of America; University of Texas Southwestern Medical Center, UNITED STATES OF AMERICA

## Abstract

Disturbances of 24-hour or circadian rhythms imposed by everyday irregular work and/or social schedules have been linked to vascular disease, including ischemic stroke. Using an established shift work-like paradigm and preclinical model for ischemic stroke, we have shown that environment-induced circadian dysregulation exacerbates stroke outcomes differentially to a greater extent in male than female rats. Because more severe stroke outcomes and circadian rhythm disturbances have been linked to gut pathophysiology, the present study examined the effects of chronic shifts in the LD cycle on gut cytoarchitecture, microbiota composition, metabolites, and inflammatory mediators for evidence of corresponding sex differences. Two independent cohorts of adult (5–7mo) rats exposed for 50d to fixed or shifted (12hr advance/5d) LD 12:12 cycles were used to examine the effects of circadian dysregulation on: fecal microbiome composition in relation to stroke survival (Cohort 1); and gut morphology, metabolites and inflammatory mediators (Cohort 2). Circadian entrainment of activity rhythms was stable during exposure to fixed LD cycles but was severely disrupted in shifted LD rats. Relative to fixed LD controls, male but not female rats exposed to shifted LD cycles were distinguished by significant alterations in the composition of the gut microbiome including reduced alpha diversity, shifts in beta diversity and correlations between the abundance of beneficial gut bacteria and stroke survival. The effects of circadian dysregulation on gut microbiota were accompanied by evidence of pathologic gut morphology (i.e., shorter and blunted villi, crypt hyperplasia, disruption of tight junction proteins and gut barrier integrity), decreased circulating levels of the neuroprotective short-chain fatty acid butyrate, and elevated serum concentrations of endotoxin and proinflammatory cytokine IL-17A in shifted LD male rats. These results suggest that alterations in gut cytoarchitecture, microbiota, metabolites and inflammatory mediators may contribute to sex differences in the effects of circadian dysregulation on ischemic stroke outcomes.

## Introduction

The generation and photoentrainment of mammalian circadian rhythms is mediated by a hierarchical organization of multiple, cell-autonomous clocks. The suprachiasmatic nucleus (SCN) of the anterior hypothalamus functions as a master pacemaker whereas peripheral clocks located in other brain regions and tissues provide for the local coordination of tissue- or cell-specific processes so as to occur at the “right time” of day or night relative to each other and to environmental time cues [1]. Dysregulation of circadian clocks throughout the body and corresponding misalignment of local tissue- and cell-specific rhythms has been frequently linked to shift work, chronic jet lag, and workplace or social influences that commonly inflict highly irregular schedules on our sleep-wake cycles, mealtimes, and other behavioral and physiological processes. In this regard, it is noteworthy that about 20–25% of the US population engage in shift work at non-traditional times during evenings, nights, or rotating shifts, and the resultant dysregulation/misalignment of circadian rhythms has been identified as a contributing element in vascular disease or related risk factors (e.g., diabetes, obesity, inflammation) in general and stroke in particular [2–10].

Because shift work is associated with other major risk factors for vascular disease, such as smoking, poor diet and lower socioeconomic status, the results of these epidemiological studies do not address the extent to which shift work-induced circadian dysregulation independently contributes to cardiovascular and stroke pathology. Our recent studies with animal models have resolved this limitation of epidemiological investigations by using chronic shifts of the light-dark (LD) cycle to determine whether shift work-like modulation of circadian rhythms alone exacerbates the severity of stroke-induced brain injury. In adult (5–7mo) rats, we found that circadian rhythm dysregulation in response to recurrent advances (12hr/5d) of the LD cycle amplifies sex differences in pathological outcomes of ischemic stroke immediately following exposure to shifted LD cycles [11]. Similar to reported epidemiological findings [2], circadian dysregulation after exposure to shifted LD cycles was acutely marked by high rates of stroke-induced mortality in males (70%), but not females (20%). At present, little is known about the mechanisms by which shift work-induced circadian dysregulation amplifies stroke severity and how this variable interacts with other non-modifiable risk factors such as biological sex to modulate the pathological effects of stroke.

Our studies and other published reports have implicated two broad and overlapping processes involving inflammation and gut-brain communication in mediating the effects of circadian dysregulation on stroke outcomes. Stroke is an inflammatory disease and post-stroke disruption of the blood brain barrier as well as activation of systemic and brain resident immune cells are key factors in the pathophysiology of stroke. Consistent with the contributions of inflammation and immune cell activation to stroke-induced brain injury and functional deficits, circadian rhythm dysregulation has been shown to promote proinflammatory responses of the innate immune system, leading to a persistent inflammatory condition [12–14]. The role of inflammation and the immune system in mediating the pathological effects of circadian dysregulation on stroke is indirectly supported by our finding that global disruption of circadian rhythms in mice with targeted clock gene mutations enhances activation of proinflammatory M1 macrophages and their inflammatory responses, thereby promoting tissue inflammation [13]. Over the past decade, increasing evidence suggests that interactions between the gut and brain play an important role in both normal and pathologic function [15]. Importantly, both stroke and the disruption of circadian rhythms have been shown to alter gut barrier integrity, microbiota composition and metabolites [16–18]. Because the gut houses large cohorts of immune cells and gut metabolites that modulate inflammation, it is possible that circadian dysregulation may exacerbate ischemic stroke severity by promoting gut pathophysiology and consequently an inflammatory condition. Using an established light-dark (LD) cycle shifting paradigm, the objective of the present study was to investigate whether the exacerbation of stroke pathology in response to controlled circadian dysregulation is linked to alterations in gut barrier integrity, microbiota and beneficial metabolites, and elevations in circulating levels of inflammatory mediators.

## Materials and methods

### Animals

Adult (5–7-month-old) female and male Sprague Dawley rats were purchased from Harlan Laboratories and maintained in the AAALAC-accredited vivarium at the Texas A&M University Health Science Center under controlled temperature (22–25°C) and lighting (LD 12:12) conditions with food (standard rat chow) and water available *ad libitum*. All animals were housed individually in cages equipped with running wheels to provide for continuous analysis of wheel-running activity. All animal experiments were performed in accordance with the National Institutes of Health Guide for the Care and Use of Laboratory Animals. Animal procedures used in this study were conducted in compliance with Animal Use Protocol # 2020−0044 as reviewed and approved by the Institutional Animal Care and Use Committee at Texas A&M University. All lab personnel were required to take general lab animal handling classes and were trained in survival surgery and euthanasia procedures by experienced senior staff in the lab.

**Fig 1:**
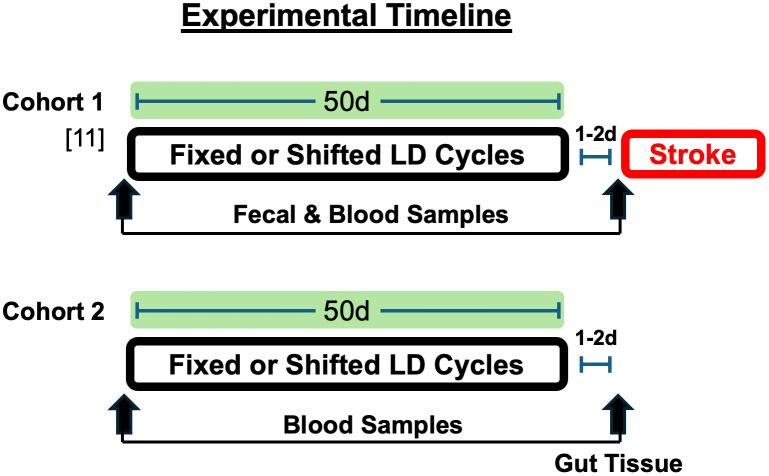
Experimental timeline. At 5-7 months of age, male and female rats were exposed for 50 days to fixed or shifted (12hr advance/5d) LD 12:12 cycles. **Cohort 1** consisted of male (@n = 16) and female (@n = 16) rats from our study examining the effects of circadian dysregulation on ischemic stroke outcomes [11]. In this cohort, microbiome composition and short-chain fatty acids (SCFAs) were analyzed in fecal and saphenous blood samples collected around mid-day (1100-1200hr) immediately before and ≈1-2 days after the conclusion of experimental LD cycle manipulations when both treatment groups were exposed to the same LD 12:12 schedule (pre- and post-treatment). Then all animals were subjected to ischemic stroke surgery at the same relative time during the circadian cycle (i.e., inactive phase; ≈ ZT 2-8), and stroke outcomes/mortality were assessed for 5 days. **Cohort 2** represents separate groups of fixed and shifted LD rats (@n = 8 males and n = 8 females) to analyze gut morphology and barrier integrity, and circulating levels of inflammatory mediators (bacterial endotoxin LPS and the proinflammatory cytokine IL-17A). The timing of blood and/or tissue sample collection (pre- and post-treatment) is indicated by the arrows (**Cohort 1**: fecal samples and blood; **Cohort 2**: blood and portion of distal ileum).

To analyze the effects of circadian dysregulation, experiments used a chronic light-dark (LD) cycle shift paradigm that has been shown to be effective in desynchronizing circadian rhythms and in inducing pro-inflammatory responses of immune cells, leading to a persistent inflammatory condition [12–14]. After baseline acclimation under standard LD 12:12 conditions (lights-on at 0600hr; light intensity = 110–170 lux at 500–580nm) for about 2 weeks, two cohorts of animals (**Cohort 1** and **Cohort 2**) were randomly divided into 2 groups and exposed for 50 days to either the same “fixed” LD 12:12 cycle or to a “shifted” LD 12:12 cycle (Fig 1). During exposure to the shifted LD paradigm, lights-on was advanced by 12hr (at 1800hr) every 5 days and these shifts in the LD cycle were repeated for 5 full cycles. At the conclusion of this treatment period (i.e., 50 days), both groups were exposed to the same standard LD 12:12 schedule (lights-on at 0600hr).

### Analysis of wheel-running activity

To confirm the long-term effects of circadian dysregulation on the rhythm of wheel-running behavior, all fixed and shifted LD rats were housed individually in cages equipped with running wheels. Wheel-running activity was continuously recorded, stored in 10-minute bins, graphically depicted in actograms, and analyzed using ClockLab data collection and analysis software (ActiMetrics, Evanston, IL). Entrainment and qualitative parameters of the activity rhythm were analyzed as described previously [11].

### Cohort 1

**Middle cerebral artery occlusion (MCAo).** Cohort 1 consisted of male (@n = 16) and female (@n = 16) rats exposed to the LD cycle shift paradigm and then subjected to ischemic stroke surgery from our original study demonstrating that circadian dysregulation exacerbates sex differences in ischemic stroke outcomes [11]. Transient occlusion of the left middle cerebral artery was performed by stereotaxic surgery and injection of endothelin-1 (ET-1) as described previously [11]. Briefly, animals were anesthetized (xylazine-15 mg/kg and ketamine-87 mg/kg), placed in a Kopf stereotaxic apparatus and maintained on heating pads during the procedure. A small clip was placed on the footpad (hindlimb) to record oxygen saturation and heart rate non-invasively. A midline incision was made on the scalp, and a craniotomy was performed on the left side with a small drill. Endothelin (ET)-1 (American Peptide Company Inc, CA; 3µl of 0.5 µg/µl) was then injected at a rate of 0.25µl per 30 seconds onto the middle cerebral artery. To minimize backflow, the syringe was maintained in place for 3 minutes following ET-1 administration. Following surgery, lidocaine (0.1 ml; 0.5%) was topically applied to the incision site and warm saline was administered intraperitoneally. Animals were constantly monitored until they recovered from anesthesia. As a precaution, small dishes of moistened food and water were placed on the floor of the cage post-operatively, and then animals were kept in a recovery room for extended care and observation for the next 48 hours. Thereafter, animals were moved down to the vivarium rooms and observed 3 times a day for the next 72 hours. All animals were paired housed to reduce distress after recovery and weighed daily to monitor loss of weight. The physical appearance and behavior of animals was carefully surveyed following experimental procedures and surgery.

Using this well-established model of MCAo-induced ischemic stroke [11,19,20,21], death is not an expected or planned outcome and most animals (≈90%) recover with some impairment of sensorimotor behavior. Endpoint criteria for euthanasia of animals experiencing adverse effects of stroke surgery are: 1) lack of appetite and/or dehydration; 2) >15% loss of body weight; and 3) hunched posture, or lethargy, Post-stroke survival was carefully recorded at 24h intervals. No animals reached endpoint criteria for euthanasia although death was observed in an unexpected number of males (see Results) upon health assessment on day 1 after surgery. All surviving animals were euthanized with sodium pentobarbital (IP, 100 mg/kg) at day 5 post-MCAo. Stroke outcomes and behavioral impairments in these animals have been reported previously [11].

**DNA Isolation, Sequencing, and Bioinformatics**. Analysis of the gut microbiome and metabolites in the circulation was performed on male and female rats from Cohort 1 that had been exposed to fixed or shifted LD cycles for 50 days [11]. In this cohort, fecal samples (pre- and post-treatment) were collected around mid-day of the LD 12:12 cycle (1100–1200hr) immediately before (baseline) and ≈1–2 days after the conclusion of experimental LD cycle manipulations when both treatment groups were exposed to the same LD 12:12 schedule (Fig 1). In addition, pre- and post-treatment saphenous blood samples for short chain fatty acid analysis (see below) were similarly collected under isoflurane anesthesia (inhalation with non-rebreathing circuit at 1–3% concentration with O_2_) from all animals in Cohort 1. After collection, serum (30–80µl) and fecal samples were frozen immediately and stored at -80^o^ C. Then all animals were subjected to ischemic stroke surgery at the same relative time during the circadian cycle (i.e., inactive phase; ≈ ZT 2–8) and stroke outcomes were assessed for 5 days. Approximately 1–2 months later, an aliquot of 100 mg (wet weight) of each fecal sample was used for DNA extraction using a MoBio Power soil isolation kit (MoBio Laboratories, USA) following the manufacturer’s instructions. A single‐step 30-cycle PCR using the HotStarTaq Plus Master Mix Kit (Qiagen) was performed using primers 515F (5′‐ GTGYCAGCMGCCGCGGTAA ‐3′) [22] to 806R (5′‐ GGACTACNVGGGTWTCTAAT ‐3’) [23] under the following conditions: 94°C for 3 minutes, followed by 28 cycles (5 cycles used on PCR products) of 94°C for 30 seconds, 53°C for 40 seconds and 72°C for 1 minute, after which a final elongation step at 72°C for 5 minutes was performed. Illumina sequencing of V4 region of the bacterial 16S rRNA genes was performed at the MR DNA laboratory (www.mrdnalab.com, Shallowater, Texas).

The demultiplexed sequences were imported into QIIME2 (v. 2024.10) for analysis. The DADA2 denoising procedure [24] was used to remove chimeric sequences and identify amplicon sequence variants (ASVs). To assign taxonomy, SILVA release 138 was used with the VSEARCH algorithm [25]. To determine phylogenetic relationships, sequences were aligned using MAFFT and a phylogenetic tree was constructed with FastTree [26]. Downstream analysis was performed in QIIME2 (v. 2024.10) and R (v. 4.5.0). A complete list of used R packages can be found in S1. The samples were then rarefied at the lowest per sample number of reads, 88,120 for the male dataset and 57,780 for the female dataset, for even diversity analysis depth. Alpha diversity was calculated using the observed features, and Shannon metrics to estimate community richness and evenness. A Shapiro-Wilk test was performed on the calculated alpha diversity metrics to assess normality. Both metrics were found to violate normality, thus a generalized linear mixed-effects models (GLMM; Gamma family, log link) were used. For all models the fixed effects were circadian condition, timepoint, and their interaction, while a random effect for animal (Animal_ID) was included to account for repeated measures (i.e., lm (alpha_diversity ~ Condition * Timepoint + (1 | Animal_ID)). Estimated marginal means (emmeans) were then calculated for the condition and timepoint interaction. Pairwise post-hoc comparisons were calculated, and the resulting p-values were adjusted for multiple comparisons with the Tukey method. Beta diversity was calculated using weighted UniFrac dissimilarity to estimate differences in overall community structure. A permutational multivariate analysis of variance (PERMANOVA) model was used to assess significance using the vegan package [27]. The PERMANOVA model tested the effects of circadian condition, timepoint, and their interaction. To account for repeated measures, permutations were constrained with a blocking factor of the Animal ID across 999 permutations. Pairwise comparisons were then performed between all circadian condition and timepoint pairs. The resulting p-values were then corrected for multiple comparisons using the Holm-Bonferroni method. Additionally, intra-individual weighted UniFrac distance was extracted from pre- and post- pairs to assess individual change in beta diversity. Resulting distances between LD conditions were compared using a Wilcoxon rank-sum test.

The MaAsLin3 (v. 1.0.0) was used to identify differentially abundant and prevalent taxa at the genus level [28]. Only genera present in at least 10% of samples at a relative abundance of 0.01% were considered for differential abundance analysis. The MaAsLin3 linear mixed-effects models were fitted to fixed and shifted LD pre- and post- pairs with Animal ID included as a random effect, to account for repeated measures. To adjust for multiple comparisons p-values were corrected within MaAsLin3 using the Benjamini-Hochberg procedure. Following correction, joint q-values < 0.05 were considered significant for all above tests. Visualizations were produced in R with the packages ggplot2 (v. 3.5.2), phyloseq (v. 1.52.0), qiime2R (v. 0.99.6), ggprism (1.0.6), and ggsci (v. 3.2.0). Pearson’s correlation coefficients were determined to analyze the relationship between the abundance of specific bacterial taxa in fecal samples and stroke survival as gauged by the number of days (1–5) that animals survived after ischemic stroke.

Raw sequencing data have been deposited in the NCBI Sequence Read Archive (SRA) and are publicly accessible under the accession number PRJNA1334100. Code used for downstream analyses of 16S rRNA gene sequencing data is available on GitHub at https://github.com/JonathanTurck02/CD_gut_analysis.

**Short chain fatty acid analysis.** Short-chain fatty acids (SCFAs) were analyzed in serum samples from animals in Cohort 1 obtained immediately before and after exposure to experimental lighting conditions. Serum samples were aliquoted (50ul) and extracted with a methanol:chloroform:water-based extraction method. Samples were spiked with 0.1 mM d7 butyric acid as an internal standard. SCFAs (butyric acid, isobutyric acid, acetic acid, valeric acid, isovaleric acid, propionic acid) were detected and quantified on a gas chromatography triple quadrupole mass spectrometer (TSQ EVO 8000, Thermo Scientific, Waltham, MA) at the Texas A&M University Integrated Metabolomics Analysis Core.

### Cohort 2

**Gut histology, Immunohistochemistry and ELISA assays.** Analysis of the effects of circadian dysregulation on gut morphology and circulating levels of LPS and inflammatory cytokines required the collection of gut tissue and blood samples from an independent cohort that was **not** subjected to ischemic stroke because stroke itself: 1) is an inflammatory disease involving the activation of microglia, recruitment of peripheral inflammatory cells and secretion of inflammatory mediators (e.g., LPS, IL-17); 2) alters gut barrier integrity [29–31]; and 3) serum volumes from saphenous blood samples obtained from rats in Cohort 1 were insufficient for assaying circulating levels of inflammatory mediators. For analysis of circadian dysregulation-induced changes in gut morphology and barrier integrity, a separate cohort of rats (**Cohort 2**) was purchased shortly after completion of experiments with Cohort 1. All animals were housed individually in cages equipped with running wheels and divided at ~5mo of age into two treatment groups exposed to fixed or shifted (@n = 8 males and n = 8 females) LD cycles as described above (Fig 1). Immediately before experimental LD cycle manipulations, saphenous blood samples were collected under isoflurane anesthesia (see Cohort 1) from all animals around mid-day of the LD 12:12 cycle (1100–1200hr). Following exposure to experimental lighting conditions (post-treatment), all animals from both treatment groups were anesthetized with sodium pentobarbital and euthanized by decapitation to provide for the collection of terminal blood samples and a portion of the distal ileum at the same time during the standard LD 12:12 cycle (1100–1200hr).

After dissection, the distal ileum was post-fixed, and embedded in Cryo-OCT compound (Leica Microsystems, Buffalo Grove, IL, USA). Embedded tissue blocks were stored at -80^o^ C until sectioning. Cryosections (10μm) were collected on glass slides, fixed in 4%PFA and stained for hematoxylin and eosin (H&E) as previously described [19,30,32,33]. The sections were visualized and photographed with the FSX100 Cell Imaging System (Olympus) at × 10 magnification. To assess structural changes in gut morphology, villus width as well as the length of the villus and crypt layers were measured from these images using the ImageJ software following established intestinal morphometric methods [19,30,32–34]. Consistent with previously published methodologies, villus dimensions were measured from the base at the crypt to its tip for villus length and at its widest point for villus width. The length of the crypt layer was measured from the base of the villus to the submucosal layer and muscularis externa. Similar morphometric approaches have been widely used to assess intestinal epithelial integrity and gut remodeling in studies examining stroke, TBI, and intestinal barrier dysfunction [19,30,34]. These measurements were assessed for all intact villi (10–15] on three sections per animal and reported as a ratio of villus length/width and villus length/crypt length. Villus length-to-width as well as villus length-to-crypt length ratios and the number of crypts per villus were subsequently calculated to assess alterations in gut architecture [19,30].

Immunofluorescence for Zonula Occludens (ZO-1) was performed as previously described [19,30,32]. Cryosections (10μm) were collected on glass slides and incubated in blocking buffer (5% bovine serum albumin (BSA), 0.1% Triton X-100 in PBS, pH 7.4) for 1 h at room temperature. The sections were then incubated overnight at 4^0^C with primary antibody to ZO-1 (rabbit polyclonal; ThermoFisher, #691–7300) at 1:1000 n PBS with 2.5% BSA. After rinses (3X) with PBS containing 0.5% Tween, slides were incubated with secondary antibody (goat anti-rabbit IgG [H + L] Alexa Fluor 555 [ThermoFisher, Catalog #31210]) at 1:2000 dilution for 1 h at room temperature. The sections were then washed three times in PBS and coverslipped with mounting media containing the nuclear dye DAPI (Fluoroshield, Abcam). Fluorescent images were acquired using the FV12-IX83 confocal microscope. The patterns of ZO-1 staining were evaluated to assess epithelial tight junction organization using approaches comparable to previously published studies examining intestinal epithelial barrier integrity and gut epithelial organization [19,30]. Quantitative analysis of the intensity of ZO-1 immunofluorescence at the brush border of intestinal villi was independently conducted by two investigators blinded to treatment groups. For this analysis, the 10X objective was used to capture images from three representative gut sections through the ileum of each animal (n = 5/treatment group), and then three randomized fields of view (FOV) with 10–15 villi were analyzed in each section. Analysis of the integrated intensity of ZO-1 immunofluorescence was performed using FIJI software (Version1.54). Before analysis, images were converted to 16-bit grayscale and binary processing was used to generate black and white images (Pixel radius: 3, mask weight: 0.6, radius: 1.0 pixel). Thresholds were manually adjusted between 15–20% depending on staining intensity. ROIs encompassing a single villus were used to measure the integrated intensity of ZO-1 staining in 10–15 different villi within each FOV.

In the same cohort used for gut histology (Cohort 2), circulating levels of the bacterial endotoxin LPS and the proinflammatory cytokine IL-17A were determined using established ELISA assays [35]. Serum LPS levels were quantitatively measured using a double-sandwich ELISA method (MyBiosource, USA) and colorimetric detection system. Serum and standard samples (100μl/well) were assayed in duplicate. Assay sensitivity ranged from 15.6–1000ng/ml. Plates were read at 450nm in a plate reader (BioTek), and sample measurements were interpolated from the standard curve. Serum levels of the cytokine IL-17A were measured in blood samples from fixed and shifted LD animals using a multiplexed magnetic bead immunoassay (Milipore Corp. MA) and a Bio-Plex suspension array system (Bio-Rad Laboratories, CA) following manufacturer’s protocols. In 1 fixed LD male rat, there was insufficient serum volume in both pre- and post-treatment samples to assay serum LPS levels so was analysis performed on remaining samples in this group (n = 7).

### Statistical analysis

Statistical analysis was performed on SCFA, IL-17A and LPS data to determine the significance of LD treatment (fixed versus shifted) and time (pre- versus post-treatment) differences using a two-way ANOVA adjusted for multiple comparisons in conjunction with Sidák’s post-hoc pairwise analysis. For gut morphology analysis, LD treatment group differences were evaluated using an unpaired *t*-test. Mann-Whitney U tests and Pearson’s correlation coefficients were determined to analyze the relationship between the abundance of beneficial gut bacteria and stroke survival. In each case, LD treatment- and/or time-related differences in circulating levels of SCFA, IL-17A and LPS, and in gut morphological features and ZO-1 immunofluorescent intensity were considered significant at p < 0.05 (GraphPad, San Diego, CA).

## Results and discussion

Stable entrainment of the circadian rhythm of wheel-running activity was observed in all animals during baseline exposure to standard LD 12:12 conditions. Consistent with the results of our previous studies [11,35], activity rhythms remained stably entrained with daily onsets of activity occurring shortly after lights-off in fixed LD rats but were severely desynchronized in the shifted LD group throughout the period of exposure to experimental lighting conditions [see 11].

**Fig 2:**
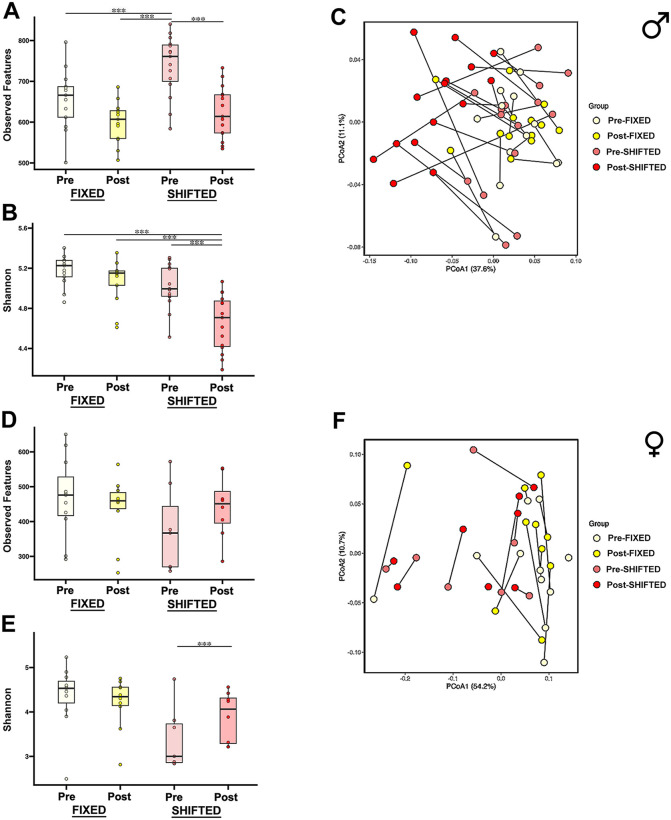
Effects of shifted LD cycles on microbiome composition. Microbial community composition in males and females maintained under fixed or shifted LD cycles. Alpha diversity, assessed as richness (Observed Features; **A**, **E**) and evenness (Shannon index; **B**, **F**), was measured at pre- and post-timepoints for each condition in males (**A**, **B**; n = 13-15/condition) and females (**E**, **F**; n = 7-11/condition). Beta diversity was calculated using weighted UniFrac principal coordinate analysis (PCoA) to assess changes in overall community structure between timepoints for males (C) and females (**G**). Weighted UniFrac boxplots of intra-individual difference in pre vs. post distances for males (**D**) and females (**H**) exposed to fixed or shifted LD cycles. ***, p < 0.001; ** p < 0.01.

16S Amplicon sequencing analysis yielded important information on treatment group differences in microbiome composition, albeit limited to the genus level and relative quantification with minimal functional characterization. The diversity of gut microbiota is thought to provide an important index of gut health and our analysis identified treatment-related differences in diversity metrics both within (alpha) and between (beta) samples. Alpha diversity metrics in male rats revealed significant circadian condition x time interactions for both richness (*F*_1,26_ = 6.38, *p* = 0.018) and evenness (χ^2^ (1)=8.02, *p* = 0.005). Post-hoc analysis revealed decreased richness (Observed ASVs metrics, Fig 2A) as well as evenness (Shannon Index, Fig 2B) in male rats in the shifted LD group (Observed ASV: *t*_26_ = −5.62, *p* < 0.001; Shannon: *z* = −5.77, *p* < 0.001) but not in fixed controls (Observed ASV: *t*_26_ = −1.78, *p* = 0.3; Shannon: *z* = −1.51, *p* = 0.4). Female rats, ins*t*ead, showed no significant differences in richness (condition x time interaction: χ^2^ (1)=3.75, *p* = 0.053). However, female rats had a significantly higher Shannon index (Fig 2F) in the shifted LD group (*z* = 3.68, *p* = 0.001) but not in fixed LD controls (*z* = −0.84, *p* = 0.84).

Along with the reduced alpha diversity, male rats exposed to shifted LD cycles showed shifts in beta diversity, presumably reflecting an overall disruption of gut health. Beta diversity in male rats, calculated through the weighted UniFrac distance matrix, showed significant differences in overall microbial community composition (PERMANOVA: *pseudo-F*_3,52_ = 7.17, *p* = 0.001, *R*_2_ = 0.29). Pairwise comparisons confirmed significant differences over time within both the shifted (*pseudo-F*_1,28_ = 11.33, *p* = 0.001) and fixed LD groups (*pseudo-F*_1,24_ = 2.03, *p* = 0.032). However, the portion of variance related to time was different, with a larger effect observed in the shifted LD group (*R*_2_ = 0.29), whereas fixed LD male rats showed high overlap (*R*_2_ = 0.08), both of which are depicted graphically in Fig 2C. Alternatively, no significant differences were observed in fixed (*pseudo-F*_1,19_ = 0.86, *p* = 0.15, *R*_2_ = 0.04) or shifted (*pseudo-F*_1,13_ = 0.62, *p* = 0.11, *R*_2_ = 0.05) LD female rats over time, as reflected by the high degree of overlap in the PCoA plots (Fig 2G). Intra-individual differences between pre- and post- distances had significantly larger change over time in shifted LD males compared to their fixed counterparts (*W* = 34, *p* = 0.003, Fig 2D). No significant change in intra-individual differences between pre- and post- distances was observed between shifted and fixed LD females (*W* = 39, *p* = 0.74, Fig 2H).

**Fig 3:**
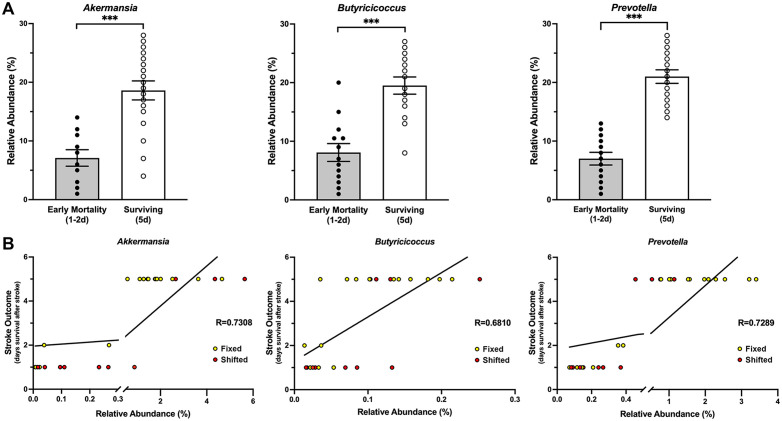
Relationship between the fecal abundance of beneficial gut bacteria and stroke survival in male rats that were exposed to fixed or shifted LD cycles. **(A)** Ranks plot of the relative abundance of: *Akkermansia* (**left**), *Butyricicoccus* (**middle**), and *Prevotella* (**right**) in fecal samples from male rats on fixed and shifted LD cycles that showed early mortality (1-2d) or survival (5d) after ischemic stroke. (***p < 0.0001; Mann-Whitney test) **(B)** Pearson correlation coefficients comparing the number of days (1-5) that male rats on fixed (n = 13) or shifted (n = 15) LD cycles from Cohort 1 survived after ischemic stroke with the relative abundance of: *Akkermansia* (**left**), *Butyricicoccus* (**middle**), and *Prevotella* (**right**) in fecal samples collected prior to stroke surgery. Symbols represent individual data values for each rat in fixed (yellow) and shifted (red) LD groups. Lines in each graph denote simple linear regression for the data set with corresponding *p* values.

**Fig 4:**
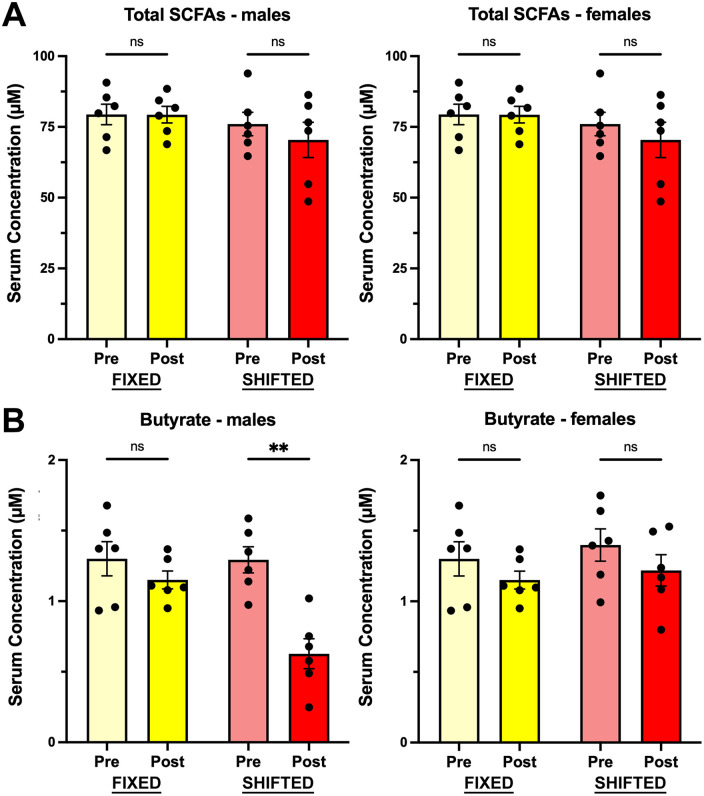
Effects of shifted LD cycles on circulating levels of short-chain fatty acids (SCFA). Serum levels of total SCFAs (**A**) and butyrate (**B**) in male (**left**) and female (**right**) rats exposed to fixed (yellow bars; n = 6) or shifted (red bars; n = 6) LD cycles. Histograms depict mean total SCFA and butyrate (µM) concentrations (+SEM) in blood collected immediately before (PRE) and after (POST) experimental LD cycle manipulations. *, p < 0.05; **, p < 0.01.

Differential abundance analysis with MaAslin3 at the genus level identified several taxa that were disparately abundant over time within fixed and shifted LD rats. Genera characterized by significant associations with the combined effects of time and circadian dysregulation are shown in Table 1 (male rats) and Table 2 (female rats). In males, pre- versus post-comparisons revealed 8 significantly associated taxa in fixed LD controls and 24 in shifted LD rats at significance level q-value joint <0.05. Of interest, post-treatment abundance of SCFA-producing taxa such as *Harryflintia* and *Prevotellaceae (UCG-001, UCG-003)* were significantly reduced in shifted LD male rats when compared to baseline levels (Table 1). Moreover, the significant decreases in post-treatment abundance of *Butyricimonas*, *Bacteroides*, and *Rikenellaceae* in shifted LD male rats are notable because some members of these taxa are considered potential pathobionts that may become pathogenic or promote inflammation under certain conditions [36]. Further analysis using Mann-Whitney U tests on combined groups of fixed and shifted LD males revealed that the abundance of *Akkermansia* (p = 0.0001), *Butyricicoccus* (p < 0.0001), and *Prevotella* (p < 0.0001) were significantly decreased in the early mortality (day 1–2) group relative to the values found in all surviving (day 5) males (Fig 3A). Moreover, Pearson correlations corroborated significant positive correlations between the abundance of several beneficial gut bacteria and stroke survival (Fig 3B). In both shifted and fixed LD males, a positive relationship was observed between stroke survival (i.e., number of days that animals survived after stroke) and the relative abundance of *Akkermansia* (p < 0.01, r = 0.7308), *Butyricicoccus* (p < 0.01, r = 0.6810), and *Prevotella* (p < 0.01, r = 0.7289). The positive associations between the abundance of these beneficial bacteria and stroke survival are noteworthy in relation to the reported functions of *Akkermansia* in maintaining gut barrier integrity and inhibiting intestinal inflammation by suppressing the production of inflammatory cytokines [37–39], *Butyricicoccus* in producing the neuroprotective SCFA butyrate [40,41], and *Prevotella* in modulating neuroinflammation [20,42,43]. In contrast, females had only 1 association with significant pre- versus post-difference in fixed LD controls and 6 in the shifted LD group. In accord with our findings that mortality was low in females and that stroke survival (in days) did not differ between treatment groups [11], no significant positive or negative correlations were observed between the abundance of these beneficial bacteria and stroke survival in shifted or fixed LD female rats. Gut microbes critically affect neural function through the production of metabolites such as SCFAs [44], which serve a variety of beneficial roles including the modulation of immune cell activation and maintenance of the blood-brain barrier and blood-gut barrier. Because butyrate is a well-known SCFA with demonstrated roles as an anti-inflammatory mediator and neuroprotectant in response to stroke [21,40], we examined whether the effects of circadian dysregulation on gut microbiome composition are coupled with a decrease in the extravasation of this beneficial gut metabolite. In both sexes, circulating levels of total SCFAs were not significantly different over time (pre- vs post-treatment; F_(1,20)_: 0.3772; p = 0.5460) in either fixed or shifted LD rats (Fig 4). In contrast, sex differences were manifested in the effects of circadian rhythm dysregulation on serum levels of butyrate. Following experimental LD treatments, serum butyrate levels in male rats were significantly decreased in the shifted LD group relative to baseline pre-treatment values (p < 0.01; t = 4.801, df = 20) but showed no significant differences in fixed LD controls when compared to basal levels (p = 0.8753; t = 1.080, df = 20). In females, post-treatment levels of butyrate were not significantly different in fixed (p = 0.8055; t = 1.009, df = 20) or shifted (p = 0.9055; t = 1.214, df = 20) LD rats when compared to baseline levels in both groups. Serum levels of the SCFAs, acetate, propionate, and valerate, in both sexes were not significantly altered over time (pre- vs post-treatment) in either fixed or shifted LD rats.

**Table 1:** MaAsLin3 differential abundance statistics for males.

	Β Coefficient	q-value	Median (%)	Range (%)
**FIXED: POST vs. PRE**				
*Increased*				
*Monoglobus*	1.21	**<0.001**	2.08	0.65–8.3
*Ileibacterium*	3.09	**<0.001**	0.01	0–3.4
*Akkermansia*	3.55	**0.005**	0.25	0.007–5.6
*Decreased*				
*Tyzzerella*	−1.65	**0.005**	0.05	0.003–0.2
*Lachnospiraceae_UCG-006*	−0.95	**0.005**	0.02	0–0.45
*Lachnoclostridium*	−0.95	**0.005**	0.17	0.044–0.76
*UBA1819*	−1.54	**0.031**	0.07	0.007–0.41
*Butyricicoccus*	−1.29	**0.023**	0.12	0–0.64
**SHIFTED: POST vs. PRE**				
*Increased*				
*Monoglobus*	1.40	**<0.001**	2.08	0.65–8.25
*Turicibacter*	1.95	**<0.001**	2.91	0.2–12.14
*Romboutsia*	1.79	**<0.001**	1.35	0.01–6.82
*Clostridium_sensu_stricto_1*	1.64	**<0.001**	3.6	0.14–12.06
*Defluviitaleaceae_UCG-011*	1.43	**<0.001**	0.02	0–0.07
*Unclassified Christensenellaceae*	0.96	**<0.001**	0.03	0.01–0.08
*Desulfovibrio*	0.98	**0.001**	0.09	0.01–0.74
*OPB41*	0.84	**0.024**	0.01	0–0.101
*NK4A214_group*	0.52	**0.005**	1.34	0.42–3.48
*[Eubacterium]_nodatum_group*	0.28	**0.044**	0.04	0.009–0.12
*[Eubacterium]_coprostanoligenes_group*	0.16	**0.048**	1.82	0.99–4.32
*Rothia*	0.68	**0.034**	0.02	0.005–0.066
*Enterorhabdus*	1.08	**0.004**	0.02	0.002–0.12
*Unclassifed Oscillospirales*	0.72	**0.015**	0.02	0.003–0.058
*Decreased*				
*Harryflintia*	−0.82	**0.024**	0.01	0–0.069
*Parabacteroides*	−1.11	**0.044**	0.28	0.059–0.98
*Unclassified Peptococcaceae*	−1.11	**0.044**	0.31	0.04–0.73
*Bacteroides*	−1.25	**0.005**	3.49	0.67–13.67
*Butyricimonas*	−1.29	**0.011**	0.11	0–0.64
*Parasutterella*	−1.94	**<0.001**	0.60	0.12–6.49
*Prevotellaceae_UCG-003*	−2.02	**<0.001**	1.08	0.07–5.36
*Prevotellaceae_UCG-001*	−2.04	**0.004**	0.11	0–0.9
*Rikenellaceae_RC9_gut_group*	−2.09	**0.005**	0.04	0–0.63
*Gastranaerophilales*	−2.32	**<0.001**	0.04	0.003–0.42

**Table 2:** MaAsLin3 differential abundance statistics for females.

Genus	β Coefficient	q-value	Median (%)	Range (%)
**FIXED: POST vs. PRE**				
*Increased*				
*Frisingicoccus*	3.32	**0.049**	0	0–0.10
*Decreased*				
*Paraprevotella*	−3.00	**0.043**	0	0–0.24
*Prevotellaceae_UCG-003*	−2.33	**0.046**	0.05	0.004–3.35
**SHIFTED: POST vs. PRE**				
*Decreased*				
*Unclassified Coriobacteriales*	−0.93	**0.002**	0.39	0.009–0.17
*Adlercreutzia*	−0.41	**0.005**	0.26	0.073–0.85
*Enterorhabdus*	−0.71	**<0.001**	0.17	0.016–0.75
*Unclassified Ruminococcaceae*	−1.31	**<0.001**	0.5	0.016–4.61

**Fig 5:**
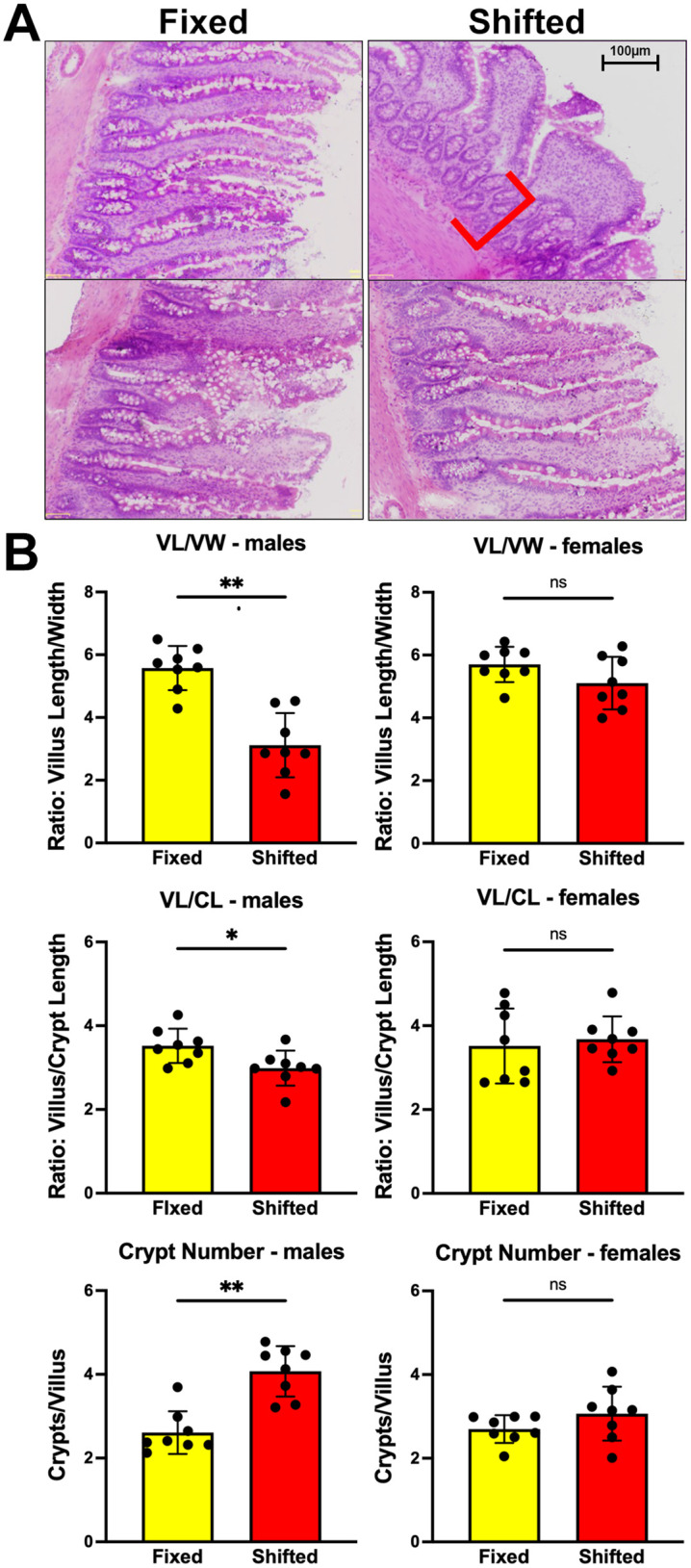
Effects of shifted LD cycles on gut morphology and barrier integrity. (**A**) H&E-stained sections of the ileum from male (**top**) and female (**bottom**) rats exposed to fixed (**left**) or shifted (**right**) LD cycles. Crypt hyperplasia and increased crypt length (red bracket) were evident in shifted LD males. Scale bar = 100µm. (**B**) Histograms depict mean (± SEM) ratios of villus length:width (**top**) and villus:crypt length (**middle**), and the number of crypts per villus (**bottom**) for each group (n = 8). *, p < 0.05; **, p < 0.01.

Histological analysis also revealed evidence of sex differences in the effects of circadian dysregulation on gut cytoarchitectural organization. In all fixed LD rats, gut morphology was characterized by the normally elongated and evenly spaced villi with a single row of crypt cells. In comparison, the distal ileum in shifted LD males, but not females, was distinguished by alterations in gross morphological appearance in which the villi were shorter, wider and blunted (Fig 5A). These decreases in villus length and width in shifted LD males effectively serve to reduce the villi surface area and thereby are thought to lower nutrient absorption in the ileum. Due to the reductions in both villus length and width, male rats exposed to shifted LD cycles were correspondingly marked by a significant decrease (p < 0.01; t = 5.598, df = 14) in the villus length/width ratio relative to their male counterparts in the fixed LD group (Fig 5B). No significant differences in the villus length/width ratio were observed between shifted and fixed LD females (p = 0.1190; t = 1.661, df = 14).

In addition to shorter, blunted villi, the gross changes in gut barrier structure in shifted LD males were accompanied by crypt hyperplasia. Crypt cells in the intestine provide stem cells that aid in repair of the epithelium as well as growth and protection of the ileum. Crypt hyperplasia was differentially observed in male, but not female, rats exposed to shifted LD cycles (Fig 5B), resulting in a significant decrease (p < 0.05; t = 2.577, df = 14) in villus to crypt length ratio as well as a significant increase in the number of crypts per villus (p < 0.01; t = 5.252, df = 14) compared to fixed LD males. This crypt hyperplasia and corresponding reduced villus to crypt length ratio are indicative of increased pro-inflammatory responses in the gut and the impaired function of tight junction proteins to maintain gut barrier integrity. Thus, circadian dysregulation had marked effects on gut morphology and barrier integrity in males, but not females, exposed to shifted LD cycles including deceases in villus length:width ratio along with crypt hyperplasia and disruption of the gut epithelial barrier.

**Fig 6:**
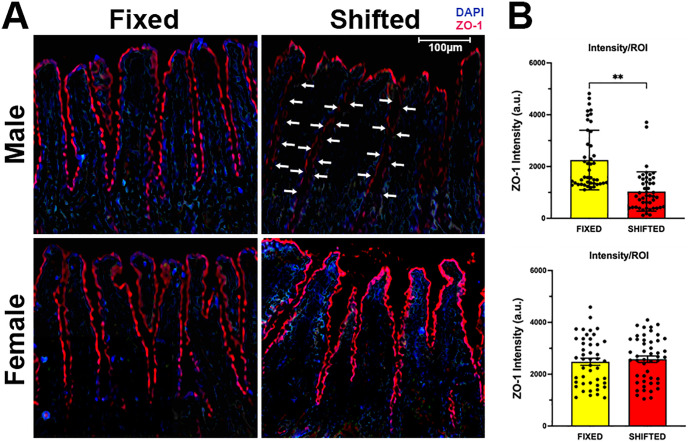
Immunofluorescent localization of the tight junction protein ZO-1 in sections (10µm) of the distal ileum. (**A**) Representative micrographs of ZO-1(**red**)-immunofluorescence in male (**left**) and female (**right**) rats exposed to fixed and shifted LD cycles. In the shifted LD male, white arrows indicate the location of the epithelial barrier where inter-epithelial ZO-1 immunofluorescence was virtually absent. Scale bar = 100µm. (**B**) Bar graphs depict quantitative analysis of the integrated intensity of ZO-1 immunofluorescence (mean ± SEM) from gut villi in fixed (n = 5) and shifted (n = 5) LD rats. For this analysis, three representative gut sections through the ileum were analyzed from each animal and three randomized FOV with 10-15 villi/FOV were captured in each section. In turn, the data points represent integrated intensity of ZO-1 immunofluorescence from 3-4 villi in the ileum that were selected as ROI. (**p < 0.01; Mann-Whitney test).

The structural integrity of the gut barrier was further examined using the pattern of immunohistochemical localization of the tight junction protein, ZO-1 at the brush border of epithelial cells lining the gastrointestinal tract. Similar to the observed gut dysmorphology in response to circadian dysregulation, this analysis demonstrates that gut barrier integrity was disrupted exclusively in male rats exposed to shifted LD cycles. ZO-1 immunofluorescence was localized contiguously at brush border of the villi in fixed LD males as well as in females from both treatment groups (Fig 6A). However, the gut barrier was distinctly altered in shifted LD male rats such that ZO-1 immunostaining was absent or greatly diminished in ≈25−35% of the villi analyzed in the ileum. Further quantitative analysis of immunofluorescent intensity confirmed subjective observations on group differences in ileal distribution of this tight junction protein as the integrated intensity of ZO-1 immunofluorescence in the villi of fixed LD males was significantly greater (p < 0.01) than that found in their shifted LD counterparts. Group comparisons of fixed and shifted LD female rats showed no significant differences in the integrated intensity of ZO-1 immunofluorescence (p = 0.5594) localized in ileal villi.

**Fig 7:**
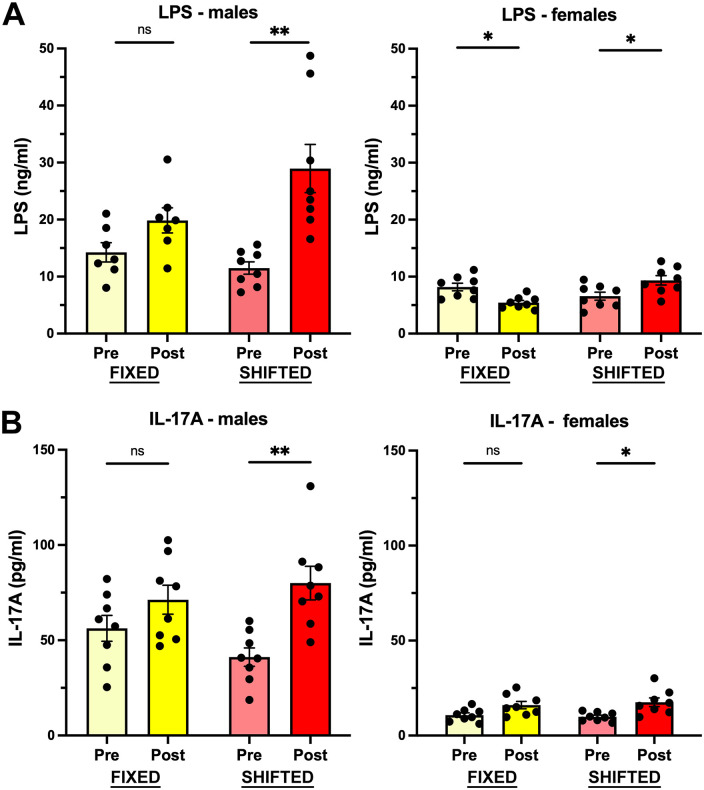
Effects of shifted LD cycles on circulating levels of lipopolysaccharide (LPS) and the inflammatory cytokine IL-17A. Serum levels of LPS (A) and IL-17A (B) in male (left) and female (right) rats exposed to fixed (yellow bars; n = 7-8 due to insufficient sample volume in 1 animal) or shifted (red bars; n = 8) LD cycles. Histograms depict mean LPS (ng/ml) and IL-17A (pg/ml) levels (+SEM) in blood collected immediately before (PRE) and after (POST) experimental LD cycle manipulations. *, p < 0.05; **, p < 0.01.

Because circadian rhythm dysregulation promotes a chronic basal inflammatory state [13,14], we next examined the effects of the shifted LD paradigm on the endotoxin LPS and cytokine IL-17A, which act synergistically to intensify proinflammatory signaling and activate immune cells [45,46]. In both fixed and shifted LD rats, serum levels of LPS (Fig 7A) and IL-17A (Fig 7B) were analyzed at baseline (pre-treatment) and immediately after exposure to experimental lighting conditions (post-treatment). In accord with previous reports [30], sex differences were observed in circulating levels of bacterial endotoxin LPS (Fig 7A) such this gut-derived inflammatory mediator was much higher in males, irrespective of treatment (fixed, shifted) and timing (pre- vs post-treatment). In both fixed and shifted LD males, post-treatment LPS levels were elevated over time (F_(1,26)_: 18.58; p = 0.0002). Planned post-hoc comparisons revealed that after experimental LD treatments serum LPS levels in male rats were significantly elevated in the shifted LD group relative to baseline pre-treatment values (p < 0.01; t = 4.777, df = 26) but showed no significant differences in fixed LD controls when compared to basal levels (p = 0.6569; t = 1.435, df = 26). In females, a significant interaction effect was observed (F_(1,28)_:17.14; p = 0.0003) between the pre- and post-treatment levels of LPS, and planned comparisons indicate that post-treatment levels of serum endotoxin were significantly increased (p < 0.05; t = 2.943, df = 28) in the shifted LD group but were significantly decreased (p < 0.05; t = 2.911, df = 28) in fixed LD controls when compared to baseline levels in both groups.

Circulating levels of IL-17A in male rats were elevated in both the LD fixed and shifted groups over time compared to their respective baseline values (F_(1,28)_:14.22; p = 0.0008) (Fig 7B). However, post-treatment IL-17A levels were significantly increased (p < 0.01; t = 3.844, df = 28) by approximately 2-fold in the shifted LD males but were not significantly different (p = 0.6169; t = 1.489, df = 28) in fixed LD controls of the same sex relative to baseline levels observed in both groups. In females, no interaction effect was observed (F_(1,28)_:0.4905; p = 0.4895) between the pre- and post-treatment levels of IL-17A, and planned comparisons indicate that post-treatment levels of this inflammatory cytokine were significantly increased in shifted (p < 0.05; t = 3.243, df = 28) LD rats, but not in fixed LD controls (p = 0.1789; t = 2.252, df = 28), relative to baseline values in each group.

Circadian dysregulation and the extent of stroke outcomes have been independently associated with alterations in the gut microbiome and barrier function. Under normal conditions, gut contents are partitioned from general circulation via a series of barrier elements, including the intestinal epithelial barrier and the blood-gut barrier (BGB) [29]. Increased permeability of the BGB not only allows transfer of gut contents but also alters the composition of resident microbes, and consequently, synthesis of beneficial gut metabolites. Disruption of circadian rhythms during exposure to constant light has been shown to increase intestinal permeability, decrease diversity of gut microbiota and dramatically downregulate beneficial butyrate-producing microbial communities [18]. In turn, gut barrier breakdown and bacterial translocation are associated with more severe stroke outcomes [16,47]. The present study provides further evidence that circadian rhythm dysregulation and stroke severity are closely interconnected through changes in gut health. It is noteworthy that sex-differences were manifested in the impact of circadian dysregulation on the gut microbiome. Male rats exposed to shifted LD cycles were distinguished by decreased alpha diversity, altered beta diversity, and significant reductions in beneficial bacterial taxa such as *Harryflintia* and *Prevotellaceae*. In contrast, female rats showed only modest microbiome alterations and no significant disruptions in overall diversity metrics. This sex divergence closely mirrors epidemiological and experimental data indicating that circadian dysregulation interacts with biological sex to influence disease susceptibility [2,11]. The observed reduction of SCFA-producing genera in males is particularly relevant, given the neuroprotective roles of SCFAs in maintaining blood–brain barrier and gut barrier integrity, regulating immune responses, and mitigating stroke-induced injury [21,40].

Exposure to shifted LD cycles produced pronounced disruptions in intestinal cytoarchitecture and barrier integrity in male, but not female, rats. Histological and immunohistochemical analyses revealed that the distal ileum in shifted LD males was marked by shortened and blunted villi, crypt hyperplasia, and disrupted or absent localization of the tight junction protein ZO-1, indicative of impaired absorptive capacity and increased epithelial permeability (‘leaky gut’). Importantly, the absence of these morphological alterations in females exposed to shifted LD cycles demonstrates a sex-specific vulnerability of the male gut epithelium to circadian dysregulation. Consistent with this compromise of gut barrier integrity, serum endotoxin (LPS) concentrations were markedly elevated in shifted LD males, whereas females exhibited only modest post-treatment changes in LPS levels. In parallel, circulating levels of IL-17A were substantially increased relative to baseline in shifted LD males but exhibited a much smaller elevation in shifted LD females. IL-17A, a cytokine produced primarily by Th17 and γδ T cells, has been shown to promote crypt cell proliferation and epithelial remodeling [46,48], consistent with the observed crypt hyperplasia in males exposed to shifted LD cycles. Moreover, IL-17A synergizes with LPS to amplify proinflammatory cascades within both the gut and central nervous system [49,50]. These findings are consistent with published reports on sex differences in the effects of changes in the gut microbiome and barrier integrity on stroke outcomes [29–31,51] and collectively support a model in which circadian dysregulation initiates a feed-forward loop in males: epithelial barrier disruption facilitates endotoxin leakage, which in turn promotes IL-17A upregulation, further exacerbating barrier dysfunction and driving a persistent systemic inflammatory state. This male-biased response of the gut–immune axis may provide a vital link between circadian dysregulation and exaggerated injury after ischemic stroke.

An unexpected finding was that small elevations in circulating IL-17A levels were observed in shifted LD females despite the absence of overt changes in gut morphology or tight junction organization. This dissociation suggests that inflammatory signaling and alterations in structural barrier integrity may not be tightly coupled in females. IL-17A is traditionally viewed as a proinflammatory cytokine; however, accumulating evidence indicates that it also participates in epithelial maintenance and tissue repair. Indeed, IL-17A has been reported to stimulate epithelial regeneration and promote barrier restoration following injury [52]. Thus, the increased IL-17A levels in shifted LD females may reflect a compensatory or protective response that contributes to preservation of gut integrity rather than barrier disruption. Furthermore, estrogen-mediated protection of the intestinal epithelium may allow females to tolerate circadian dysregulation-induced immune activation without developing the gut morphological and microbiome abnormalities distinguishing shifted LD males. Although a modest contribution of age- and time-dependent changes cannot be excluded, the small, but significant, elevation of IL-17A in shifted LD females suggests that circadian dysregulation elaborates this immune response. This relative preservation of gut barrier integrity and microbiome composition in females, despite increased inflammatory signaling, highlights a potential mechanism underlying their relative resilience to circadian dysregulation and stroke-related pathology.

Taken together, our findings suggest that circadian rhythm dysregulation may amplify stroke severity via gut–brain axis interactions involving changes in gut microbiome composition and barrier integrity, altered SCFA metabolism, and systemic inflammation. Importantly, these changes are more pronounced in shifted LD males, aligning with our prior observations of higher stroke mortality in circadian-dysregulated males compared to females [11]. Beyond stroke, the convergence of circadian misalignment, gut dysfunction, and inflammation has broad relevance to human health, as shift work has been linked to elevated risk for cardiovascular disease, diabetes, obesity, cancer, and Alzheimer’s disease-related dementias [10]. Our data provide further evidence for these associations, implicating sex-specific gut pathophysiology as a potential driver.

While this study identifies compelling sex differences in gut and immune responses to circadian dysregulation, several limitations warrant consideration. First, although we observed correlations between microbial taxa abundance and stroke survival, causality cannot be inferred. Future studies employing microbiome transfer or targeted microbial supplementation will be critical to establish whether restoring beneficial taxa (e.g., *Akkermansia*, *Butyricicoccus*, *Prevotella*) can mitigate circadian dysregulation-induced stroke severity. Another limitation is that comparison of microbiome composition and SCFA levels with gut morphology and inflammatory mediators required independent analysis of two separate cohorts of animals because stroke itself promotes inflammation and alters gut barrier integrity [29–31]. This independent analysis of separate cohorts presumably provides a reliable “snapshot” in time of parallel effects of circadian dysregulation but certainly tempers mechanistic interpretations of our data. Furthermore, we focused on the ileum, but circadian rhythm dysregulation may differentially affect other intestinal segments. Likewise, even though our gut histological and immunohistochemical data demonstrate that the structural integrity of the gut barrier was altered in response to shifted LD cycles, further analysis using functional assays (e.g., FITC-dextran gavage) is necessary to corroborate corresponding changes in barrier function. In addition, we acknowledge that exercise may have some implications in the sex-dependent effects of circadian rhythm dysregulation on stroke outcomes because: 1) exercise associated with running wheel activity improves stroke outcomes [53,54]; and 2) sex-, but not, treatment-dependent differences in the total amount of daily wheel-running behavior were observed in our previous stroke study [11] such that daily activity levels were approximately 10-fold greater in female subjects than in their male counterparts within each treatment group. Finally, although our model simulates rotating shift work, it does not capture lifestyle variables such as diet, stress, or socioeconomic factors that often accompany to circadian misalignment in individuals where workplace or social influences commonly impose highly irregular schedules on sleep-wake patterns, mealtimes and other health-related processes.

## Conclusions

In summary, this study demonstrates that controlled circadian dysregulation differentially perturbs gut architecture, microbiome composition, metabolite extravasation, and inflammatory signaling in a sex-dependent manner, with males showing greater vulnerability. These gut-mediated changes may play a role in mediating the effects of circadian dysregulation on stroke outcomes and other chronic diseases linked to shift work. The gut–brain axis thus represents a critical target for interventions aimed at mitigating the adverse health effects of circadian misalignment.
